# Co-infection with Campylobacter and rotavirus in less than 5 year old children with acute gastroenteritis in Nepal during 2017–2018

**DOI:** 10.1186/s12887-020-1966-9

**Published:** 2020-02-13

**Authors:** Vishnu Bhattarai, Saroj Sharma, Komal Raj Rijal, Megha Raj Banjara

**Affiliations:** 1 Central Department of Microbiology, Tribhuvan University, Kirtipur, Kathmandu, Nepal; 2 Kanti Children’s Hospital, Maharajgunj, Kathmandu, Nepal

**Keywords:** *Campylobacter*, Rotavirus, Co-infection, Diarrhoea, Children

## Abstract

**Background:**

Diarrhoea, although easily curable, is a global cause of death for a half million children every year. Rotavirus and *Campylobacter* are the most common etiological agents of diarrhoea in children less than 5 years of age. However, in Nepal, these causative agents are not routinely examined for the diagnosis and treatment. The main objective of this study was to determine *Campylobacter* co-infection associated with rotavirus diarrhoea in children less than 5 years of age.

**Methods:**

A cross-sectional study was conducted at Kanti Children’s Hospital (KCH), Kathmandu, Nepal from November 2017 to April 2018. A total of 303 stool specimens from children affected with diarrhoea were processed to detect rotavirus using a rapid rotavirus antigen detection test kit, and *Campylobacter* by microscopy, culture and biochemical tests. Antibiotic susceptibility tests of *Campylobacter* isolates were performed according to European Committee on Antimicrobial Susceptibility Testing (EUCAST) guidelines 2015.

**Results:**

Of 303 samples, 91 (30.0%) were positive for co-infection with rotavirus and *Campylobacter*. Rotavirus mono-infection was detected in 61 (20.1%), and *Campylobacter* mono-infection was detected in 81 (26.7%) samples. Patient’s age, month of infection, untreated water and frequent soil contact were the major risk factors for infections. Clinical features such as > 9 loose motions per day, fever, vomiting, mild to moderate dehydration, diarrhea persisting 6–9 days and presence of mucus in stool were significant (*p* < 0.05) clinical features, and were more severe in coinfection compared to mono-infections in multivariate analysis.

**Conclusion:**

The study shows a high rate of rotavirus and *Campylobacter* coinfection in children with diarrhoea. Diagnosis based management of diarrhoeal cases can guide the specific treatment.

## Background

Diarrhoea remains a serious health burden in children less than 5 years of age in developing countries. Globally, diarrhoea kills around 525,000 children less than 5 years of age each year [1]. The commonest etiological agents of acute watery diarrhoea in young children in developing countries are rotavirus, enterotoxigenic *Escherichia coli, Shigella* spp.*, Campylobacter jejuni* and *Cryptosporidium parvum* [2].

Viruses are primary agents of diarrhoea during the winter in developed countries whereas bacteria are the main agents of diarrhoea in rainy season in developing countries [3]. However, rotavirus is found to be a single dominant enteric pathogen among children in most of the developed and developing countries [4]. The number of deaths attributable to rotavirus infection and associated disease in children younger than 5 years in 2008 was estimated to be 453,000 (95% CI 420,000-494,000) [5]. With the introduction of rotavirus vaccine, the number of deaths in children less than 5 years of age declined from 528, 000 in 2000 to 215, 000 in 2013; and decreased the percentage of hospitalization due to acute gastroenteritis caused by rotavirus [6, 7].

World Health Organization recommended integrating rotavirus vaccine into national immunization program [8, 9]. Although the incidence of rotavirus infection among children in developed and developing countries is similar, outcomes often vary widely with 82% of fatalities estimated to occur in developing countries [6, 10]. Rotavirus infections are an important cause of hospitalization, causing significant economic impact on poor countries [11]. Studies published on rotavirus infection in Nepal from 1999 to 2007 showed rotavirus positivity rates ranged from 17 to 39% among all hospitalized children less than 5 years [12–15]. Various published studies from 2008 to 2017 reported different prevalence rates of rotavirus infections in diarrhoeal children, ranging from 22 to 53% [16–19].

Studies revealed that co-infection does exist between enteric bacteria and viruses [20, 21]. This evidence collectively demonstrates that co-infection by bacterial and viral pathogens play a critical role in disease progression. Infectious diseases cause most of the child deaths in developing countries [22], but the etiological agents are usually unknown and can lead to overuse/misuse of antibiotics, which may exacerbate the antibiotic resistance, already a global threat [23]. This study focuses on co-infection of *Campylobacter* in rotavirus infected children and explores associated risk factors and clinical features.

## Methods

A hospital based cross-sectional study was conducted from November 2017 to April 2018. A total of 303 stool samples were collected from Kanti Children’s Hospital (KCH), Kathmandu, Nepal. Written informed consents and clinical and demographic information were obtained from guardians/caretakers of the patient. Samples were collected from children less than 5 years of age, presenting with diarrhoea as reported by parents in hospital. The samples were tested for rotavirus using Onsite rotavirus Ag Rapid Test kit (CTK Biotech, Inc. San Diego, USA) for rapid diagnosis of rotavirus, and the samples were taken in the laboratory for the detection of *Campylobacter* causing infection by culture on *Campylobacter* blood-free selective agar base supplemented with Campy blood free selective medium (Charcoal cefoperazone deoxycholate agar, CCDA) Selective Supplement (SR0155, containing cefoperazone and amphotericin B antibiotics) (Thermo-Fischer, Oxoid, UK). The inoculated medium was incubated at 37 °C and 42 °C in microaerophilic condition for 24 to 48 h using Campy gas pack (Oxoid, UK). After incubation, colonies appeared colorless or grey and spread like droplets. A presumptive diagnosis was made by wet mount preparation for darting motility. The isolated bacteria were identified based on the morphological character of the colonies, Gram staining of the isolate (Staining was performed with the application of carbol fuchsin as gram counter stain for 5 min), oxidase test, catalase test and sensitivity to nalidixic acid (30 μg) to differentiate *Campylobacter* spp. from other Enterobacteriaceae. Hippurate hydrolysis test was performed for differentiation of *Campylobacter* spp. A total of 4 days was required for confirmed diagnosis of infection with *Campylobacter*. Modified Kirby-Bauer Disc diffusion technique was used for testing the susceptibility pattern of different isolates towards various classes of antibiotics in Mueller Hinton Agar (MHA) with 5% defibrinated sheep blood. Antibiotics were used according to EUCAST guidelines (2015). Data analysis was done using IBM Statistical Package for Social Sciences version 20.0 (SPSS, Inc., Chicago, IL, USA). The association among co-infection and mono-infection with rotavirus and *Campylobacter* was tested using the Chi-square test for differences in proportions; and logistic regression analysis was used to assess the association between infection and the risk factors. A *p*-value less than0.05 was considered statistically significant.

## Results

The study included three hundred and three diarrhoeal children less than 5 years of age during the study period. The highest number of patients (*n* = 118) were from age group 7–12 months. There were 207 (68.3%) male and 96 (31.7%) female patients.

### Detection rate of pathogens

**Fig. 1:**
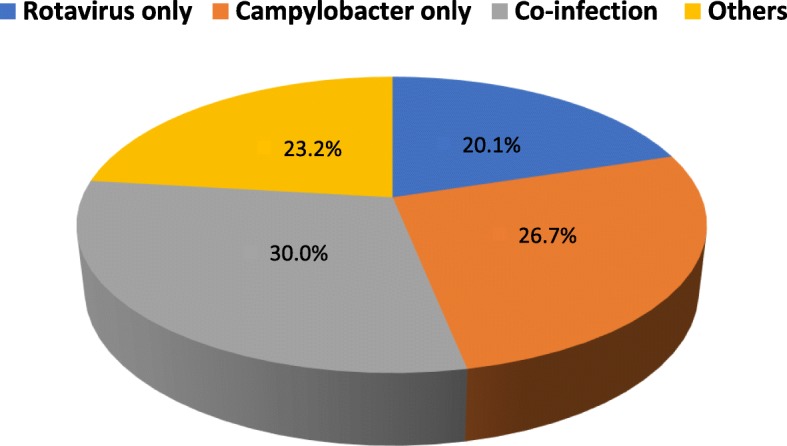
Prevalence of *Campylobacter* and rotavirus mono- and co-infections and other infections, Nepal, 2017/18. *Other means Other than rotavirus and *Campylobacter* infection

The study was focused on detection of rotavirus and *Campylobacter* spp., and at least one of these pathogens were detected in 233 (76.9%) samples. Among 303 children with acute watery diarrhea, rotavirus mono-infection was detected in 61 (20.1%), *Campylobacter* mono-infection was detected in 81 (26.7%), co-infection was detected in 91 (30.0%) (Fig. 1).

### Age wise distribution of different infections in children

The highest number of rotavirus mono-infection was detected in 7–12 months age group category which accounted 29 (47.5%) of total rotavirus mono-infection. Similarly, highest number of *Campylobacter* mono-infection 34 (42.0%) was found in the < 6 months age group. The co-infection was observed highest (35, 38.5%) in 7–12 months of age (Table 1). Distribution of different infections in children less than 5 years was not statistically significant (χ^2^test, *p* > 0.05).

**Table 1:** Age-related distribution of *Campylobacter* and rotavirus mono- and co-infections

Age (months)	Type of infection			
	Rotavirus only n (%)	*Campylobacter* only n (%)	Co-infections n (%)	Undetected n (%)
< 6	14 (23.0)	34 (42.0)	26 (28.6)	19 (27.1)
7–12	29 (47.5)	29 (35.8)	35 (38.5)	25 (35.7)
13–24	10 (16.4)	7 (8.6)	19 (20.9)	13 (18.6)
25–36	3 (4.9)	6 (7.4)	7 (7.7)	5 (7.1)
37–60	5 (8.2)	5 (6.2)	4 (4.4)	8 (11.4)
Total	61 (100.0)	81 (100.0)	91 (100.0)	70 (100.0)

### Risk factors for different infections in children

In multivariate analysis, infection in February was associated with a decreased risk of rotavirus mono-infection [adjusted odds ratio (AOR) = 0.26, 95% CI = 0.07–0.98, *p* = 0.047] than in November. Except for the age group of 25–36 months, all age groups were significantly associated with decreased risk of *Campylobacter* mono-infection compared to < 6 months age. No soil contact (AOR = 0.06, 95%CI = 0.01–0.47, *p* = 0.008) was significantly associated with a decreased risk of *Campylobacter* mono-infection compared to frequent soil contact. Infection in January (AOR = 11.34, 95%CI = 1.27–101.27, *p* = 0.030) and February (AOR = 25.32, 95%CI = 2.68–238.69, *p* = 0.005) were significantly associated with a higher risk of co-infections compared to November (Table 2).

**Table 2:** Risk factors for Rotavirus and *Campylobacter* mono-infection and co-infection in multivariate analysis

Risk factors	Rotavirus monoinfection		*Campylobacter* monoinfection		Co-infection	
	AOR (95%CI)	*P* -value	AOR (95%CI)	*P*-value	AOR (95%CI)	*P*-value
Months						
November	1		1		1	
December	0.59 (0.15–2.30)	0.451	5.57 (0.56–54.98)	0.141	8.98 (0.94–85.86)	0.057
January	0.39 (0.11–1.30)	0.127	6.06 (0.68–53.96)	0.106	11.34 (1.27–101.27)	0.030^a^
February	0.26 (0.07–0.98)	0.047^a^	5.36 (0.58–48.94)	0.137	25.32 (2.68–238.69)	0.005^a^
Age (in months)						
< 6	1		1		1	
7–12	1.70 (0.39–7.33)	0.471	0.19 (0.04–0.90)	0.037^a^	1.26 (0.32–4.87)	0.738
13–24	1.64 (0.31–8.49)	0.553	0.07 (0.01–0.42)	0.004^a^	1.42 (0.31–6.45)	0.649
25–36	3.28 (0.23–45.27)	0.374	0.10 (0.01–1.21)	0.070	0.89 (0.09–8.20)	0.925
37–60	6.04 (0.33–107.97)	0.221	0.04 (0.01–0.73)	0.029^a^	0.47 (0.03–5.74)	0.556
*Sex*						
Male	1	–	1		1	
Female	1.09 (0.52–2.26)	0.817	0.90 (0.42–1.90)	0.791	1.59 (0.80–3.12)	0.179
Parent’s occupation						
Agriculture	0.40 (0.13–1.21)	0.107	1.09 (0.31–3.86)	0.886	2.62 (0.90–7.66)	0.077
Service	0.55 (0.15–1.96)	0.359	1.30 (0.32–5.29)	0.707	1.92 (0.53–6.90)	0.318
Business	0.82 (0.24–2.82)	0.759	1.35 (0.36–4.96)	0.651	0.70 (0.21–2.34)	0.569
Others	1		1		1	
Breast feed						
No	0.30 (0.04–2.11)	0.230	1.63 (0.34–7.72)	0.534	1.18 (0.29–4.74)	0.814
Yes	1		1		1	
*Water source*						
Non-pipe borne	1		1		1	
Pipe borne	0.70 (0.27–1.75)	0.448	0.95 (0.39–2.30)	0.924	1.00 (0.45–2.22)	0.997
Drinking water						
Untreated	1		1		1	
Boiled	0.81 (0.28–2.28)	0.695	0.64 (0.21–1.94)	0.438	1.47 (0.55–3.89)	0.436
Filtered	0.24 (0.03–1.56)	0.137	0	0.998	4.45 (0.92–21.33)	0.062
Chlorinated	0.70 (0.22–2.22)	0.550	0.78 (0.25–2.46)	0.681	1.45 (0.50–4.16)	0.487
Soil contact						
No soil contact	1.86 (0.35–9.84)	0.460	0.06 (0.01–0.47)	0.008^a^	3.02 (0.62–14.73)	0.171
Infrequently	1.39 (0.51–3.75)	0.510	0.49 (0.18–1.33)	0.162	1.01 (0.41–2.42)	0.990
Frequently	1		1		1	
Hygiene						
Poor	1		1		1	
Good	0.45 (0.16–1.24)	0.125	0.97 (0.33–2.80)	0.964	1.01 (0.40–2.55)	0.973
Parent’s education level						
Illiterate	0	0.999	0	0.999	0	0.999
Literate	0.57 (0.19–1.72)	0.324	0.78 (0.24–2.53)	0.687	2.12 (0.72–6.22)	0.169
Higher level	1		1		1	

*AOR* Adjusted odds ratio, 95% *CI* 95% confidence interval, 1 = Reference, *P* < 0.05 was considered significant,^a^ = significant

### Clinical features in different infections with diarrhea

Among children with diarrhoea, clinical presentations were as follows: 23.8, 49.2 and 27.1% cases had 3–6, 7–9 and > 9 loose motions/day respectively. 64.7% cases had fever, and 85.8% had vomiting. 75.2% had no dehydration, 23.8% had mild to moderate dehydration and 1% had severe dehydration. 31.4% cases had abdominal pain. 35.6% had diarrhoea for less than 3 days, 45.9% had diarrhoea for 3–5 days, 17.5% had diarrhoea for 6–9 days, and only 1% had diarrhoea for more than 9 days. 70.6% of patients were from Out Patient Department (OPD) and only 29.4% were from In Patient Department (IPD). 60.7% had mucus in the stool and 30.0% had pus cells in the stool.

Abdominal pain and presence of pus cells in stool were less common features, which were significantly associated with rotavirus mono-infection in multivariate analysis. Pus cells in stool was common clinical feature, while fever and vomiting were less prevalent but significantly associated with *Campylobacter* mono-infection in multivariate analysis. More than nine loose motions per day, fever, vomiting, and presence of mucus in stool were most striking clinical features. Mild to moderate dehydration were less common compared with no dehydration but were significantly associated with co-infections in multivariate analysis (Table 3).

**Table 3:** Clinical features in different infections in multivariate analysis

Clinical features	Rotavirus only			*Campylobacter* only		Co-infection	
	AOR (95%CI)		*p*-value	AOR (95%CI)	*p*-value	AOR (95%CI)	*p*-value
Stool/day							
3–6	2.60 (0.79–8.55)		0.115	1.42 (0.47–4.22)	0.526	0.06 (0.01–0.23)	< 0.001
7–9	1.57 (0.56–4.38)		0.385	0.98 (0.38–2.52)	0.971	0.33 (0.13–0.85)	0.021
> 9	1			1		1	
Fever							
No	1.39 (0.69–2.79)		0.355	2.07 (1.12–3.83)	0.020	0.25 (0.12–0.50)	< 0.001
Yes	1			1		1	
Vomiting							
No	0.51 (0.16–1.57)		0.241	4.48 (1.99–10.09)	< 0.001	0.26 (0.07–0.88)	0.005
Yes	1			1		1	
Dehydration							
No-minimal	1			1		1	
Mild-moderate	2.53 (0.98–6.52)		0.053	1.22 (0.47–3.15)	0.675	0.29 (0.12–0.73)	0.009
Severe	0		0.999	0	0.999	0	0.999
Abdominal pain							
No	2.53 (1.16–5.52)		0.019	1.41 (0.75–2.62)	0.279	0.52 (0.27–1.01)	0.051
Yes	1			1		1	
Duration of diarrhea (in days)							
< 3	1			1		1	
3–5	0.87 (0.41–1.87)		0.737	1.02 (0.52–1.96)	0.952	0.66 (0.33–1.31)	0.241
6–9	0.67 (0.24–1.89)		0.458	0.54 (0.21–1.41)	0.211	1.68 (0.67–4.16)	0.262
> 9	0		0.999	2.15 (0.13–33.69)	0.584	0	0.999
Mucus							
Absent	1.39 (0.74–2.61)		0.399	0.81 (0.45–1.49)	0.514	0.46 (0.25–0.86)	0.016
Present	1			1		1	
Pus							
Absent	7.26 (2.47–21.36)		< 0.001	0.45 (0.25–0.81)	0.008	0.81 (0.43–1.50)	0.508
Present	1			1		1	

*AOR* Adjusted odds ratio, 95%*CI* 95% confidence interval, *NA* Not applicable, 1 = Reference

### Antibiotic resistance patterns in *Campylobacter* spp.

**Fig. 2:**
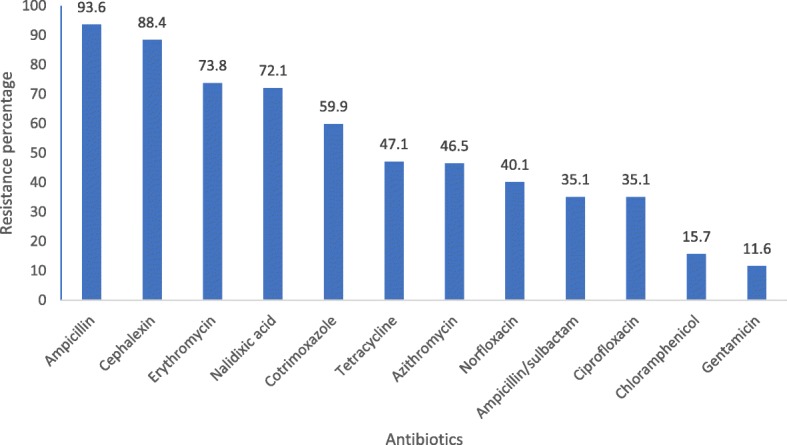
Antibiotic resistance patterns in isolated *Campylobacter* spp. (*n* = 172)

A total of 172 *Campylobacter* isolates were tested for antibiotic susceptibility. Among them, resistance to different antibiotics were: ampicillin (93.6%), cephalexin (88.4%), erythromycin (73.8%), nalidixic acid (72.1%) and cotrimoxazole (59.9%). Resistance to ampicillin/sulbactam, norfloxacin, azithromycin and tetracycline were 35.1, 40.1, 46.5 and 47.1% respectively. *Campylobacter* isolates were resistant to gentamicin (11.6%), chloramphenicol (15.7%) and ciprofloxacin (35.1%) (Fig. 2).

## Discussion

This hospital based cross sectional study explored the association between *Campylobacter* and rotavirus infections, possible risk factors and specific clinical features. In this study, most cases of acute gastroenteritis were infants. Past studies have echoed with our study that shows that diarrhea incidence peaks at age 6–11 months [24–26].

Our study identified that a higher frequency of diarrhoea was seen in males consistent with other studies [13, 16, 27, 28]. Males’ preponderance to develop diarrhoea can be explained by their increased susceptibility to outdoor physical activities thus exposure to unhygienic surroundings and flood-water during rainy seasons. In addition, it could be due to simply increased presentation of male patients at the hospital more than females [29].

*Campylobacter* and rotavirus co-infections were responsible for the one third of acute gastroenteritis cases among children less than 5 years of age visiting at the OPD and IPD clinic/vaccination unit at the KCH in Kathmandu, Nepal during November 2017–April 2018. This study confirmed previous studies where *Campylobacter* co-infection with rotavirus in children were high in less than 5 years of age [30, 31].

*Campylobacter* spp. isolation in diarrhoeal cases was high. *Campylobacter* is not included in the routine diagnosis protocol thus obviates the routine laboratory investigations on the important causative agents of diarrhoea in Nepal. *Campylobacter* was most prevalent in February. Rotavirus mono-infection was detected in one-fifth of the children, highest in the age group: 7–12 months and was consistent with other studies in Nepal and South-East Asian countries [19, 32], however, findings contrasted with few other studies from Nepal [16, 18]. It appears that infants less than 6 months of age were initially protected against severe diarrhoea, to some extent, by maternal antibodies and they seem to have acquired adequate immunity between 12 and 16 months of age [16].

Age, sex, breast feeding, water source, quality of drinking water, hygiene and education level of parents were not found to predict rotavirus mono-infection in these children. While the month of diarrhoeal illness does possess a risk for rotavirus mono-infection, a higher infection during January–February contradicts with one past study [19]. In our study, age group was the significant predictor of *Campylobacter* mono-infection. No soil contact was associated with a reduced risk of Campylobacteriosis compared to frequent soil contact. In Nepal, *Campylobacter* is not included in routine microbiological testing in patients with diarrhoea. Conventional culture of *Campylobacter* is time consuming and hence affects the management of diarrhoeal cases. Therefore, use of rapid sensitive molecular techniques could be useful for timely management of Campylobacteriosis [33].

In multivariate analysis, clinical picture of children with co-infection was more severe compared to mono-infection for most clinical signs taken into examination that included fever, vomiting, abdominal pain, duration of illness, hospitalization, frequency of loose motion/day and presence of mucus in stool. Our observations are consistent with a study among Korean children [34]. We observed that especially with rotavirus and *Campylobacter* co-infections, there was an increase in the episodes of loose motions per day, which is consistent with a study in Odisha, India [32]. A study reported from China revealed synergistic effect due to co-infection in severe childhood diarrhoea [35].

Increasing antimicrobial drug resistance by *Campylobacter* limits the number of therapeutic options, which makes empirical treatment more difficult. High proportions of antibiotic resistant *Campylobacter* isolates in our study reveals either there is antibiotic pressure or transmission of resistant bacteria from foods of animal origin [36].

## Conclusion

In conclusion, this hospital-based cross-sectional study highlights the burden of rotavirus, *Campylobacter* and co-infections in childhood diarrhoea in Nepal. Rotavirus and *Campylobacter* associated co-infections were found to be high in this study. *Campylobacter* spp., which are generally not screened for in diarrheic patients in Nepal, should also be suspected and considered into the routine diagnosis protocol. Diagnosis of the right causative agents among possible multiple infectious etiologies can help to better manage the acute childhood diarrhoea.

## Data Availability

All data pertaining to this study are within the manuscript.

## Abbreviations

Ag: Antigen
AOR: Adjusted odds ratio
CCDA: Campy blood free selective medium, Charcoal cefoperazone deoxycholate agar
CI: Confidence interval
EUCAST: European committee on antimicrobial susceptibility testing
IPD: In patient department
KCH: Kanti children’s hospital
MHA: Mueller hinton agar
OPD: Out patient department
OR: Odds Ratio

## References

[1] World Health Organization. Diarrhoea, fact sheet. 2017. Available from: http://www.who.int/topics/diarrhoea/en. Accessed on: 20 June, 2019.

[2] Platts-Mills JA, Babji S, Bodhidatta L, Gratz J, Haque R, Havt A (2015). Pathogen-specific burdens of community diarrhoea in developing countries: a multisite birth cohort study (MAL-ED). Lancet Glob Health.

[3] Mackenjee MKR, Coovadia YM, Coovadia HM, Hewitt J, Robins-Browne RM (1984). Aetiology of diarrhoea in adequately nourished young African children in Durban. S Afr Ann Trop Paediatr.

[4] Nyaga MM, Jere KC, Esona MD, Seheri ML, Stucker KM, Halpin RA (2015). Whole genome detection of rotavirus mixed infections in human, porcine and bovine samples co-infected with various rotavirus strains collected from sub-Saharan Africa. Infect Genet Evol.

[5] Tate JE, Burton AH, Boschi-Pinto C, Steele AD, Duque J (2012). Parashar UD; WHO-coordinated global rotavirus surveillance network. 2008 estimate of worldwide rotavirus-associated mortality in children younger than 5 years before the introduction of universal rotavirus vaccination programmes: a systematic review and meta-analysis. Lancet Infect Dis.

[6] Tate JE, Burton AH, Boschi-Pinto C, Parashar UD (2016). World Health Organization-coordinated global rotavirus surveillance network. Global, regional, and National estimates of rotavirus mortality in children <5 Years of age, 2000–2013. Clin Infect Dis.

[7] Burnett E, Jonesteller CL, Tate JE, Yen C, Parashar UD (2017). Global impact of rotavirus vaccination on childhood hospitalizations and mortality from diarrhea. J Infect Dis.

[8] Troeger C, Khalil IA, Rao PC, Cao S, Blacker BF, Ahmed T (2018). Rotavirus vaccination and the global burden of rotavirus diarrhea among children younger than 5 years. JAMA Pediatr.

[9] Jonesteller CL, Burnett E, Yen C, Tate JE, Parashar UD (2017). Effectiveness of rotavirus vaccination: a systematic review of the first decade of global postlicensure data, 2006-2016. Clin Infect Dis.

[10] Bonkoungou IJ, Damanka S, Sanou I, Tiendrébéogo F, Coulibaly SO, Bon F (2011). Genotype diversity of group a rotavirus strains in children with acute diarrhea in urban Burkina Faso, 2008–2010. J Med Virol.

[11] Bourdett-Stanziola L, Jiménez C, Ortega-Barria E (2008). Diversity of human rotavirus G and P genotypes in Panama, Costa Rica, and the Dominican Republic. Am J Trop Med Hyg.

[12] Pun SB, Nakagomi T, Sherchand JB, Pandey BD, Cuevas LE, Cunliffe NA (2007). Detection of G12 human rotaviruses in Nepal. Emer Infect Dis.

[13] Shariff M, Deb M, Singh R (2003). A study of diarrhoea among children in eastern Nepal with special reference to rotavirus. Indian J Med Microbiol.

[14] Sherchand JB, Haruki K (2004). Rotavirus diarrhoea in children and animals of urban and rural Nepal. J Nepal Health Res Counc.

[15] Uchida R, Pandey BD, Sherchand JB, Ahmed K, Yokoo M, Nakagomi T (2006). Molecular epidemiology of rotavirus diarrhea among children and adults in Nepal: detection of G12 strains with P [6] or P [8] and a G11P [25] strain. J Clinic Microbiol.

[16] Sherchand JB, Nakagomi O, Dove W, Nakagomi T, Yokoo M, Pandey BD (2009). Molecular epidemiology of rotavirus diarrhea among children aged < 5 years in Nepal: predominance of emergent G12 strains during 2 years. J Infect Dis.

[17] Pandey B D, Pun S B (2012). Trends of Rotavirus in Nepal. Kathmandu University Medical Journal.

[18] Sherchan JB, Ohara H, Sherpa K, Sakurada S, Gurung B, Tandukar S (2011). Rotavirus nosocomial infection in children under 5 years of age: a preliminary study in Nepal. J Nepal Paedtr Soc.

[19] Dhital S, Sherchand JB, Pokhrel BM, Parajuli K, Shah N, Mishra SK (2017). Molecular epidemiology of rotavirus causing diarrhea among children less than five years of age visiting national level children hospitals. Nepal BMC Pediatr.

[20] Calvo C, Gallardo P, Torija P, Bellón S, Méndez-Echeverría A, del Rosal T (2016). Enterovirus neurological disease and bacterial coinfection in very young infants with fever. J Clinic Virol.

[21] Grimprel E, Rodrigo C, Desselberger U (2008). Rotavirus disease: impact of coinfections. Pediatr Infect Dis J.

[22] The United Nations Children's Fund (UNICEF) (2014). Levels & Trends in Child Mortality. Report; Estimates Developed by the UN Inter-agency Group for Child Mortality Estimation.

[23] Pokharel S, Raut S, Adhikari B (2019). Tackling antimicrobial resistance in low-income and middle-income countries. BMJ Glob Health.

[24] Naficy AB, Abu-Elyazeed R, Holmes JL, Rao MR, Savarino SJ, Kim Y (1999). Epidemiology of RVA diarrhea in Egyptian children and implications for disease control. Am J Epidemiol.

[25] Amer MA, Abdel Salam SM, Ibrahim HA, Farag MA (2007). Detection of group a Rota virus and characterization of G type among Egyptian children with diarrhea. Egyptian J Med Microbiol.

[26] Fischer Walker CL, Perin J, Aryee MJ, Boschi-Pinto C, Black RE (2012). Diarrhea incidence in low- and middle-income countries in 1990 and 2010: a systematic review. BMC Public Health.

[27] Klein EJ, Boster DR, Stapp JR, Wells JG, Qin X, Clausen CR (2006). Diarrhea etiology in a children's hospital emergency department: a prospective cohort study. Clin Infect Dis.

[28] Moyo SJ, Gro N, Matee MI, Kitundu J, Myrmel H, Mylvaganam H (2011). Age specific aetiological agents of diarrhoea in hospitalized children aged less than five years in dares salaam. Tanzania BMC Pediatr.

[29] Salim H, Karyana PG, Sanjaya-Putra GN, Budiarsa S, Soenarto Y (2014). Risk factors of rotavirus diarrhoea in hospitalized children in Sanglah hospital, Denpasar: a prospective cohort study. BMC Gastroenterol.

[30] Rajendran P, Babji S, George AT, Rajan DP, Kang G, Ajjampur SS (2012). Detection and species identification of *Campylobacter* in stool samples of children and animals from Vellore, South India. Indian J Med Microbiol.

[31] Zhu XH, Tian L, Cheng ZJ, Liu WY, Li S, Yu WT (2016). Viral and bacterial etiology of acute diarrhea among children under 5 years of age in Wuhan, China. Chin Med J.

[32] Shrivastava AK, Kumar S, Mohakud NK, Suar M, Sahu PS (2017). Multiple etiologies of infectious diarrhea and concurrent infections in a pediatric outpatient-based screening study in Odisha. India Gut Pathogens.

[33] Liu KC, Jinneman KC, Neal-McKinney J, Wu WH, Rice DH (2017). Simultaneous identification of *Campylobacter jejuni*, *Campylobacter coli*, and *Campylobacter lari* with smartcycler-based multiplex quantitative polymerase chain reaction. Foodborne Pathog Dis.

[34] Koh H, Baek SY, Shin JI, Chung KS, Jee YM (2008). Coinfection of viral agents in Korean children with acute watery diarrhea. J Korean Med Sci.

[35] Zhang J, Duan Z, Payne DC, Yen C, Pan X, Chang Z (2015). Rotavirus-specific and overall diarrhea mortality in Chinese children younger than 5 years: 2003 to 2012. Pediatr Infect Dis.

[36] Ghimire L, Singh DK, Basnet HB, Bhattarai RK, Dhakal S, Sharma B (2014). Prevalence, antibiogram and risk factors of thermophilic *Campylobacter* spp. in dressed porcine carcass of Chitwan, Nepal. BMC Microbiol.

